# Community Environment Perception on Depression: The Mediating Role of Subjective Social Class

**DOI:** 10.3390/ijerph18158083

**Published:** 2021-07-30

**Authors:** Liqin Zhang, Lin Wu

**Affiliations:** 1 School of Sociology, Wuhan University, Wuhan 430072, China; zhangliqin-psy@shzu.edu.cn; 2 Department of Psychology, Shihezi University, Shihezi 832000, China; 3 Psychological Application Research Center, Shihezi University, Shihezi 832000, China

**Keywords:** community environment, subjective social class, depression, internal mechanism

## Abstract

Depression has become a major social issue of global concern, which has seriously threatened the quality of an individual’s life. Although the relationship between community environment and depression has aroused heated debate, the empirical research on the relationship between community environment perception and public depression is still relatively insufficient. Data for this study are from China Family Panel Studies in 2016, which were conducted by the institute of Social Science Survey, Peking University. This paper has tested group differences in the influence of community environment on public depression, as well as the mediating role of subjective social class between community environment perception and depression, so as to further explore the social psychological effect of community environment. The empirical study found that there are group differences in the impacts of community environment perception on depression. Specifically, men, rural residents, and people aged 60 and under are more likely to be depressed which were affected by the perception of community environment. Furthermore, we have found that the subjective social class can partly mediate community environment perception and depression. That is to say, the perception of community environment can induce depression by influencing the individual’s subjective social class. Among them, community living environment and community public facilities have the greatest impact, community emotional attachment and community security situation have the second impact, and neighborhood mutual aid and neighborhood relationship have the least impact. In other words, the community environment is deeply endowed with a social psychological effect. To ameliorate the public’s depression, it is necessary to consider the construction of community physical environment and the cultivation of harmonious community culture as powerful measures not to be ignored. In short, the important role of community environmental intervention in alleviating the public’s depression caused by social class cognition deserves attention.

## 1. Introduction

Mental disorders have become a major social issue of global concern. At present, depression has become one of the most widespread mental disorders, already presenting a worsening trend year by year and seriously threatened people’s life quality [1]. The etiology of depression has been listed as a priority in academic circles. The researchers have found that there were demographic differences in depression [2]. For example, gender, education level, employment status, living style, social activities are significantly related to depression [3], and rural female elderly is the high incidence group of depression [4,5]. At the same time, depression is closely related to the individual’s psychological factors [6,7]. Depression is often accompanied by low mood, mental retardation, cognitive impairment, sleep disorders, and other physical symptoms. Low mood and cognitive impairment are related to poor psychosocial function, which is the core performance of depression [8]. Meanwhile, recent studies pay more attention to the influence of external physical environmental factors such as residence, location, climate change, living conditions, etc. [9,10]. The external environment directly related to human activities is widely regarded as an important predictor of physical and mental health [11,12,13], which always threatens people’s emotional health [14]. Additionally, cognitive function development [15] seriously damages their mental health [16], and even aggravates depression [17]. Therefore, it is an indisputable proposition that the objective reality environment indirectly affects the human experience and behavior through individual subjective perception. Although there may be a gap between the objective environment and the public’s actual feelings, it turns out that the individual’s perception on environment is a significant predictor of depression [18]. There is no doubt that people’s social identity perceived from the environment is a factor that cannot be ignored in inducing depression [19].

As the basic field of modern residents’ life, community environment and human welfare are closely intertwined. Community environment refers to the degree of overall environment quality and appropriate degree to people’s physical and mental health development. For example, the perception of community environment is measured by the respondents’ cognitive evaluation of various elements of the community environment [20]. It is found that the community environment has a direct impact on people’s life satisfaction, subjective well-being, and mental health [21,22]. In other words, community environment factors affect the development of an individual’s cognitive function [15], which is closely related to the individual mental health [23]. Community environment is an important factor which has seriously threatened the public’s emotional health [24] and might induce depression [25,26]. Higher satisfaction with a community’s objective environment led to higher sense of coherence and lower levels of depression [27]. For example, community environmental factors such as a lack of public space and facilities in a community can trigger depression [28]. In addition to the influence of community construction environment, community environment associated with social capital such as social interaction and social relationships can effectively alleviate the depression caused by income inequality [29]. This shows that the poor perception of community environment might induce negative cognition, and especially the negative impact on depression deserves deep attention. Therefore, Hypothesis 1 is proposed in this study.

**Hypothesis** **1** **(H1).** *People with worse perception of community environment are facing more serious depression.*

How people perceive their social status is considered a predictor of physical and mental health [18]. Subjective social class refers to the relative social and economic status perceived by people, which is the result of subjective comparison and judgment [30], which has a significant predictive effect on negative emotions and can strongly predict the individual’s mental health [31]. Research evidences have shown that the individual’s social class is closely related to the internalization of behavioral problems [32]. Low social status groups have experienced more life shocks than high social status groups, resulting in more mental health inequalities [33]. In addition, according to an academic study, the influence of family socioeconomic status on the individual’s depression also cannot be ignored [34]. Previous research has found that a high level of social status, positive residence perception, and moderate life stress can promote positive cognitive aging [35], while improvements in cognitive function can significantly reduce the risk of depression [36]. For example, high socioeconomic status and superior living environment (neighborhood problems, social capital, and social participation) can effectively reduce health inequality by 40% and poor health behavior by 16% [37]. It seems very likely that subjective indicators of social relations are more likely to predict depression than objective indicators, and social isolation is more likely to lead to depression in the context of threatening rather than affirming a meaningful social identity [38]. Subjective social class is a subjective indicator of the individual’s social identity and social stratification. There is no doubt that the public’s subjective positioning of their own social class through social comparisons may induce depression and exacerbate mental health inequality. Therefore, we propose the following hypothesis.

**Hypothesis** **2** **(H2).** *People in lower subjective social class are facing more serious depression.*

The influence of community environment on individual depression has been well demonstrated by previous studies. For example, housing equity inequality worsens social trust [39] and divides social classes [40]. The negative mental health problems caused by negative social comparison can be greatly alleviated by the cross-class interaction of social relations and cultural capital in the community [41]. Research has shown that cognitive ability has a positive mediating effect, and higher subjective social class can effectively improve the individual’s mental health [42]. Therefore, the influence of community environment on the individual’s depression is worth further discussion. First, widespread environmental inequality in communities coexists with the growing economic inequality [29,43]. Socioeconomic inequality in the community is a predictor of mental health [44], which may add more social and psychological attributes to the community environment. Secondly, class differences are regarded as the factor most closely related to economic inequality [45]. Moreover, subjective social class, a better predictor of health outcomes than objective social class [46], is strongly associated with depression [47]. Third, differences in social class may lead to inequalities in public mental health [48], which is a common determinant of depression in different social contexts [49]. Subjective social class is the social stratification perceived by individuals through social comparison, which has a significant psychological effect of social isolation. Compared with life stress events, people seem to be particularly sensitive to social forms of stress (such as rejection or conflict) [19]. For example, perceived social isolation leads to social isolation and social withdrawal, which reduces social contact [50] and induces depression [38]. There is no doubt that the public’s perception of the community environment might induce depression by subjectively positioning their own social class through social comparison. Hence, Hypothesis 3 is proposed in this study.

**Hypothesis** **3** **(H3).** *Subjective social class plays a mediating role between community environment perception and depression*.

As far as we know, although the relationship between community environment and mental health has been well confirmed in previous literature, there is still no further research to explore the impact of subjective perception of community environment on public depression. In conclusion, community environment not only has a physical spatial effect, but also has certain psychological and social benefits, which may be more conducive to understanding the possible path of public depression under different social, cultural, economic, and political backgrounds. The social psychological effect of perceived community environment can also induce public depression. To address these gaps, the present study explores the intrinsic mechanism of community environment and social cognition related to depression. In order to explore the influence of community environment on depression, this study aims to introduce a new perspective to explain the mechanism. Specifically, using the data from the 2016 China Family Panel Studies (CFPS) and regression estimation method, we empirically investigate the mediating effect of subjective social class between community environment perception and depression. The perception of community environment affects the public’s subjective social class, thus enhancing the scope and intensity of public’s social comparison. People with poor perception of community environment are more likely to be dissatisfied with their social class and more likely to suffer from depression.

## 2. Materials and Methods

### 2.1. Data and Sample

Data used in the current study were obtained from the 2016 China Family Panel Studies (CFPS), a continuous large-scale nationwide survey conducted by the National Survey Research Center of China, which employed a stratified multistage probability proportional to the size sampling design and covered 25 provinces across mainland China, by tracking and collecting data at the individual, family, and community levels. The CFPS 2016 consists of adult questionnaires and children’s questionnaires, which covered society, economy, population, education, health, social relations, cognition, and attitude in China, and basically reflected the panorama of family economic and social life in China and were well representative of the whole country. Considering the complexity and comprehensiveness of environmental quality perception and subjective class perception, this study only used the adult survey, including 33,296 adults aged 16 and above. After sorting the data and eliminating the missing important variables, 4997 effective samples were selected. Data are available through the Institute of Social Science Survey Data Archive website.

### 2.2. Variables and Instrument

Community environment perception was an independent variable. Referring to previous studies, community environment is defined as community happiness index, neighborhood problems, neighborhood interaction time [23], community natural environment, community culture and entertainment activities, community safety, community roads, and public facilities [51]. In this study, community environment perception was measured by a 5-point Likert scale, including community public facilities, community living environment, community public security, community neighborhood relationship, community neighborhood mutual-assistance and community emotional attachment. A higher score corresponds to worse perception of community environment. A question (“what is the overall situation of public facilities such as education, medical treatment and transportation in your neighborhood?”) was selected, which reflected the perception of community public facilities in this study. A question (“what is the situation of the surrounding environment of your residential area, such as noise pollution, garbage stacking, etc.?”) was selected, which reflected the perception of community living environment in this study. A question (“what is the security situation around your residential area?”) was selected, which reflected the perception of community public security in this study. A question (“what do you think of your overall neighborhood relationship?”) was selected, which reflected the perception of community neighborhood relationship in this study. For these four dimensions, respondents need to score the degree of answer from “very good” to “very bad”, which assigned a value from 1 to 5 successively. For the perception of community neighborhood mutual-assistance, a question (“if you need the help of neighbors, do you think someone will help you?”) was used to represent in this study. Respondents could rate the extent of the answer from “must have” to “definitely not”, and assign 1 to 5, respectively. A question (“do you have any feelings for your community?”) was selected, which reflected the perception of community emotional attachment in this study. Respondents could rate the extent of the answer from “very affectionate” to “very unsentimental”, which assigned a value from 1 to 5 successively. The Cronbach’s alpha coefficient for the scale was 0.706 in this study.

Subjective social class was a mediating variable, which was measured by the Likert 5-point rating scale. A question (“what is your social status in local area?”) was selected, which reflected subjective social class in this study. Respondents were asked to rank the degree of the choice from “very high” to “very low”, which assigned a value from 1 to 5 successively. A higher score represented a lower subjective class perception.

Depression was a dependent variable. The Center for Epidemiologic Studies Depression Scale had been widely used as an effective method to measure individual depression, which contained 16 questions to measure negative feelings (such as “I’m worried about some small things”) and four questions to measure positive feelings (such as “I don’t feel inferior to others”). Respondents could answer “hardly (less than a day)”, “sometimes (1–2 days)”, “sometimes often (3–4 days)”, and “most of the time (5–7 days)”, which assigned a value from 1 to 4 successively to represent the responses of negative emotion items. While the response assignment of positive emotion items was scored reversely. The total CES-D score was from 0 to 80 theoretically and a higher score represented more serious depression. The Cronbach’s alpha coefficient for the scale was 0.848 in this study.

Covariates must be controlled in the process of data analysis, due to their well associations with the public’s mental health, especially depression or due to their potential to confound the relationship between community environment perception and depression. The related control variables included age, gender, household register, health condition, marital status, nature of work, and education. According to the needs of data analysis in this study, the original data were further processed. In terms of age, the values of 0, 1, and 2 were separately assigned to age groups 16–44, 45–59, and over 60, respectively. In terms of gender, “female” was assigned a value of 0 and “male” a value of 1. In terms of household registration, “rural-registered residence” a value of 0 and “city-registered residence” was assigned a value of 1. In terms of health condition, “general” and “unhealthy” were assigned a value of 0 and “relatively healthy”, “very healthy”, and “extremely healthy” a value of 1. In terms of marital status, we assigned “unmarried” and “cohabitation” a value of 0, “married with spouse” a value of 1, and “divorced” and “widowed” a value of 2. In terms of nature of work, we assigned “agricultural work” a value of 0, “non-agricultural work” a value of 1, and “jobless” a value of 2. In terms of education, we assigned “primary level” a value of 0, “intermediate level” a value of 1, and “high level” a value of 2. Illiterate, semi illiterate, and primary school were collectively referred to as primary level education, which was assigned a value of 0. Junior middle school, senior middle school, technical school, and professional high school were collectively referred to as intermediate level education, which was assigned a value of 1. Junior college, undergraduate degree, master degree, and doctor degree were collectively referred to as high level education, which was assigned a value of 2.

### 2.3. Statistical Analysis

Before statistical analysis, the original data are filtered. The data with three standard deviations higher or lower than the average score and the missing values were eliminated. The SPSS software (version 25.0, IBM Corp, Armonk, NY, USA) was used for descriptive statistics and correlation analysis.

We used the stepwise linear regression analysis to test the mediating effect of subjective social class between community environment perception and depression with maximum likelihood estimators and 95% bias-corrected confidence intervals (CIs) using 5000 bootstrapped samples repeatedly. Specifically, model 4 in the SPSS macro process plug-in developed by Hayes [52], is used to test the mediating role of subjective social class between community environment perception and depression, by strictly controlling covariates, such as age, gender, household register, health condition, marital status, nature of work, and education.

## 3. Results

### 3.1. Descriptive Analysis of Social Demographic Characteristics

As shown in Table 1, there are 4997 valid samples including 2376 males (47.50%) and 2621 females (52.50%), whose average age was 47.91 (SD = 16.753). Of the total 4997 respondents, 2376 were male participants (47.50%) and 2621 were female participants (52.50%). There are 1312 owned urban household register (26.30%) and 3685 owned rural household register (73.70%). In terms of nature of work, it can be seen that 1090 jobless participants account for 21.80%, and it is noted that the agricultural work group (40.90%) is much more than the non-agricultural work group (37.30%). Furthermore, 582 participants were unmarried (11.60%), 4021 participants were married with a spouse (80.50%), and 394 participants were divorced or widowhood (7.90%). With regards to their health condition, 3244 were healthy (64.90%) and 1753 were unhealthy (35.10%). In addition, 1413 people (28.30%) had a primary-level education, 2379 people (47.60%) had an intermediate-level education, and 1205 people (24.10%) had a high-level education.

### 3.2. Preliminary Analysis

The descriptive statistics and correlation coefficients for the study variables are presented in Table 2. Correlation analyses indicate that depression is significantly positively correlated with community environment perception (r = 0.216, *p* < 0.01) and subjective social class (r = 0.181, *p* < 0.01). Moreover, subjective social class is significantly positively associated with all dimensions of community environment perception. These results provide good preliminary support for the hypotheses. In addition, depression is also significantly positively associated with all dimensions of community environment perception. These results provided a good preliminary support for the hypotheses.

### 3.3. Group Differences in Community Environment Perception on Depression

This study showed that community environment perception had a significant positive impact on public depression (β = 0.534, *p* < 0.001). On this basis, we used linear regression to test the group differences of community environment perception on public depression (as shown in Table 3).

Firstly, we discussed the impacts of community environment perception on public depression of different genders (models 1 and 2). The results showed that, compared with women, male depression was more likely to be affected by the perception of community environment (female: β = 0.479, *p* < 0.001; male: β = 0.597, *p* < 0.001). In contrast, male depression was significantly higher than female in all dimensions of community environment. The results showed that the perception of community environment improved male depression to a certain extent.

Secondly, the difference of community environment perception on depression among different registered residences (models 3 and 4). We found that compared with urban residents, rural residents were more likely to be affected by the perception of community environment (rural: β = 0.561, *p* < 0.001; city: β = 0.516, *p* < 0.001). Urban residents were more likely to be depressed by community public facilities (rural: β = 0.944, *p* < 0.001; urban: β = 1.024, *p* < 0.001) and community living environment (rural: β = 0.944, *p* < 0.001; urban: β = 1.024, *p* < 0.001) than rural residents. However, rural residents were more susceptible to community public security (rural: β = 1.389, *p* < 0.001; urban: β = 1.047, *p* < 0.001), community neighborhood relationship (rural: β = 1.736, *p* < 0.001; urban: β = 1.484, *p* < 0.001), community neighborhood mutual aid (rural: β = 1.845, *p* < 0.001; urban: β = 1.360, *p* < 0.001), community emotional attachment (rural: β = 1.632, *p* < 0.001; urban: β = 1.406, *p* < 0.001) than urban residents. The results showed that community environment perception improved the depression of rural residents to some extent. Rural residents’ depression were more affected by community public safety, community neighborhood relationship, community neighborhood mutual assistance, and community emotional attachment, while urban residents’ depression were more influenced by the community public facilities and community living environment. In other words, the impacts of community environment perception on depression were different between rural residents and urban residents.

In addition, we discussed the different impacts of community environment perception on public depression across age (models 5–7). We found that the depression of the public aged 45–59 were the most affected by community environment perception (β = 0.655, *p* < 0.001), followed by the public aged 16–44 (β = 0.611, *p* < 0.001), and the least affected were the public aged 60 and over (β = 0.515, *p* < 0.001). The same change trends were also consistent in community public facilities, community living environment, community public safety, and community emotional attachment. However, in the dimension of community neighborhood relationship, the public aged 45–59 were the most affected (β = 1.957, *p* < 0.001), followed by the public aged 60 and over (β = 1.757, *p* < 0.001), and the public aged 16–44 were the least affected (β = 1.594, *p* < 0.001). In the dimension of community neighborhood mutual assistance, the public aged 60 and over were the most affected (β = 2.053, *p* < 0.001), followed by the public aged 44–59 (β = 1.776, *p* < 0.001), and the public aged 16–44 were the least affected (β = 1.474, *p* < 0.001). The results showed that community environment perception has greatly improved the depression of the public aged 60 and under to some extent. The depression of the public aged 60 and under were more affected by community public safety, community neighborhood relationship, community neighborhood mutual assistance, and community emotional attachment. While, the depression of people aged 60 and over were more affected by the perception of community neighborhood relationship, community neighborhood mutual assistance, and community emotional attachment.

### 3.4. The Mediating Effect of Subjective Social Class

This study showed that community environment perception had a significant positive effect on the public’s depression. On this basis, we further explored the mediating role of subjective social class between community environment perception and depression, and the comparative results of mediating effects, as shown in Table 4.

First of all, community environment perception has a significant positive effect on depression (β = 0.532, *p* < 0.001, CI = [0.462, 0.602]), and hypothesis 1 is verified. Community environment perception has a significant positive effect on subjective social class (β = 0.048, *p* < 0.001, CI = [0.038, 0.058]), and hypothesis 2 is verified. Meanwhile, subjective social class also has a significant positive effect on depression (β = 0.609, *p* < 0.001, CI = [0.395, 0.823]). Community environment perception affects subjective social class, which reaches 5.8% explanatory power. The influence of community emotional attachment on the individual’s subjective social class has reached 5.1% explanatory power, the other five dimensions of community environment perception have also exceeded 4% explanatory power. This suggests that subjective social class plays a mediating role between community environment perception and depression, which accounts for 5.45% of the total effect. This indicates that the influence of community environment perception on depression is partly due to the role of subjective social class, and community environment perception can explain 16.80% variation of depression. Accordingly, the worse the perception of community environment, the more the severity of public depression.

Similarly, we examine the mediating effect of subjective social class between each dimension of community environment perception and depression in turn, showing that the mediating effects are all significant, and hypothesis 3 is verified. Community public facilities perception can explain 13.40% variation of depression, accounting for 7.09% mediating effect. Community living environment perception can explain 13.50% variation of depression, accounting for 9.15% mediating effect. Community public security perception can explain 14.10% variation of depression, accounting for 6.67% mediating effect. Community neighborhood relationship perception can explain 14.60% variation of depression, accounting for 5.29% mediating effect. Community neighborhood mutual-assistance perception can explain 14.70% variation of depression, accounting for 5.50% mediating effect. Community emotional attachment can explain 14.80% variation of depression, accounting for 6.82% mediating effect.

In addition, results indicate that the contributions of each dimension of community environmental factors leading to public depression are different. The top two contributing factors are community living environment perception, community public facilities perception, both more than 7% explanatory power. Then, the following two contributing factors are community emotional attachment and community public security perception, both more than 6% explanatory power. While the last two contributing factors are community neighborhood mutual-assistance perception and community neighborhood relationship perception, both more than 5% explanatory power.

Community public facilities perception, community living environment perception, and community public security perception can be thought of as community physical environment perception. While, community neighborhood relationship perception, community neighborhood mutual-assistance perception, and community emotional attachment can be understood as community emotional social support perception. It can be seen that community emotional social support perception has a greater impact on individual depression than community physical environment perception. However, the mediating effect of subjective social class is just the opposite. That is to say, community physical environment perception has greater impacts on depression by the mediation of subjective social class.

## 4. Discussion

Using nationally representative data, the current research attempts to explore the impact of community environment perception on public depression and the internal mechanism from the perspective of subjective social class, and contributes to the literature through the socio-psychological effects of the community environment. The results of the study found that the perception of community environment affects the public’s depression through the mediating effect of subjective social class. Our research has drawn three valuable findings by establishing a mediating effect model.

One of our key findings is that perception of the community environment can predict depression. Good perception of the community environment means lower level of depression, which is more conducive to the individual’s mental health. The main social psychological mechanism of inducing the individual’s depression is the subjective perception induced by the spatial physical characteristics of the community, which also shows that the social and cultural factors of the living environment are the internal mechanism of inducing the individual’s psychological effects. The four dimensions, community security, community-neighbor relationship, community-neighbor mutual assistance, and community emotional attachment, have the greatest direct impact on individual depression, all exceeding 14% explanatory power. Perceived disharmony in the community and few opportunities for social interaction can also affect public depression [53]. It can be seen that community emotional support is of great significance to the social psychological mechanism that directly induces individual depression, which shows that community cultural construction is an internal mechanism that induces individual psychological effects. At the same time, the two dimensions of community public facilities and community living environment have a slightly smaller effect on public depression, but both exceed 13% of the explanatory power. There is no doubt that the physical environment of the community may also directly induce public depression. The results of this study also confirmed that men, rural residents, and people aged 60 and under are more likely to be depressed affected by the perception of community environment than the others. This is consistent with the previous study, that is to say, living conditions [40], living environment, and community environment are closely related to residents’ depression [54]. We believe that this result can be reasonably explained, since emotions are the result of the individual’s subjective perception and have contextual socio-psychological effects. In addition, economic inequality is an important factor leading to the inequality of people’s living environment. People with better economic conditions prefer to live in communities with abundant public facilities, good public security, beautiful construction environment, and harmonious interpersonal relationships, which in turn further differentiates the individual’s socio-economic status [55]. Living in the community with higher satisfaction of its environment is more conducive to promoting the individual’s mental health [29,56], which further shows that the subjective positive perception of community living environment can protect people from the negative psychological impact of social and economic inequality. Based on these findings, it is necessary for future research to pay more attention to the promotion of community environment on potential positive psychological effects.

The second major finding of this study is that the perception of community environment can predict the individual’s subjective social class. The physical characteristics of community can induce the individual’s subjective cognition. The better the perception of community environment, the higher the subjective orientation of social class, the more conducive to the formation of positive social cognition. Community environment affects the individual’s subjective social class, and the explanatory power reaches 5.8%. Among the six dimensions of community environment, only the dimension of community emotional attachment has an explanatory power of 5.1%, while the other five dimensions have less than 5%, but more than 4%. These results indicate that community environment can induce the individual’s subjective social cognition, and the community environment that promotes social activities and interpersonal relationships may be related to the lower income inequality of patients with depression [29]. We have reason to believe that the psychological effect caused by community environment is an important incentive to promote the individual’s social cognition. With the expansion of China’s urbanization and higher education, economic development, and the continuous improvement of education level, people’s demand for improving the quality of life is more and more intense, and the inequality of social wealth is greatly aggravated. People prefer to choose a superior community environment to show their success and wealth [40]. To a certain extent, a good community environment is an important external sign of success and wealth, which may lead to the improvement of personal social capital and social status [57]. It can be seen that housing wealth has become the standard for people to identify with the subjective social class, representing the symbol of identity [58]. Due to the close relationship between social resources and interpersonal relationships, the social benefits of community environment are amplified and the community environment is further endowed with positive symbolic and psychological significance. To a certain extent, community environment seems to be the symbol of social identity and the embodiment of subjective social stratification. Therefore, the superior community environment may help improve people’s subjective social class and enhance their positive social cognition [59]. The improvement of subjective social class also enhances people’s social identity, which is more conducive to promoting people to expand the scope of social interaction network, thus increasing the channel of people’s positive social interaction, and helping to alleviate people’s depression.

Finally, we find that the subjective social class partially mediates depression and community environment perception, and the mediating effects on all dimensions are significant. In other words, the perception of community environment indirectly affects the public’s depression through influencing their subjective social class. The same is true for all dimensions of community environment. First of all, the physical environment of the community living environment and community public facilities have the largest indirect effect on depression through the subjective social class (both with more than 7% explanatory power), which show that the construction of community physical space environment is the primary indicator to measure people’s perception of community environment. The higher the quality of public space and facilities, the more conducive to promoting people’s mental health [28]. Secondly, community emotional attachment and community public security are the second important factors that affect the individual’s subjective social class and depression (all over 6% of the explanatory power). The needs for community emotion attachment and residential safety come after the needs of community physical environment. It can be said that community emotional attachment and community security can also improve people’s satisfaction with the community environment, help enhance the public’s subjective social class, and promote their mental health. Thirdly, the last two factors of indirect effect are community neighborhood mutual aid and community neighborhood relationship (both with more than 5% explanatory power), which show that emotional needs are indispensable indicators to measure the public’s perception of community environment. Relatives, friends, and neighbors are closely linked through frequent social communication governed by mutually beneficial cultural rules, which regulate the formation of people’s social network and the establishment of social emotions [60]. The results are consistent with Maslow’s hierarchy of needs theory, that is, people’s low-level material needs such as survival and security needs gradually rise to high-level spiritual needs such as belonging and love needs, and finally reach the peak of self-realization of life pursuit. It is worth noting that the mediating effect of subjective social class between the perception of community living environment and depression is the largest (9.15%, only one is more than 9%). It also shows that community living environment has a great effect on depression through influencing the public’s subjective social class. The effective improvement of community living environment such as noise pollution and garbage stacking will greatly improve the public’s subjective evaluation of their own social class, and indirectly alleviate their depression. By optimizing the allocation of community green space resources [61], improving the allocation of public space and facilities [28], and building a harmonious neighborhood relationship [60], we can effectively improve the subjective social class of individuals, promote people’s mental health, and form a positive concept of social equity [33]. On the other hand, the sense of control is closely related to depression [62]. Community environment can lead to individual depression by reducing the subjective social class, which indicates that the subjective social class has the psychological effect of sense of control. This further shows that social factors perceived in the community environment can induce individual depression [63].

Our results not only inspire our understanding of this problem in the context of social culture, but also enrich the internal mechanism of the relationship between community environment and depression. In this study, we use limited variables to evaluate the attributes of community environmental factors. Unfortunately, there is no more abundant and reliable data to test these mediating mechanisms of other socio-cultural factors, such as relative deprivation, “face effect”, and positive social comparison. Therefore, it will be an important research topic in the future. Meanwhile, these results strongly support the effectiveness of community environmental intervention in reducing public depression. The results strongly suggest that cognitive optimization can effectively improve the individual’s social and psychological functions [64]. It is a necessary policy consideration to promote social cohesion by strengthening the construction of community environment and culture, so as to alleviate the public health burden caused by depression and mental health inequality. We hope that these findings will encourage the formation of relevant policies and pay enough attention to the important role of community environmental intervention in alleviating public depression caused by social class cognition.

## 5. Conclusions

In the current study, we find that community environment has an important impact on depression through situational psychosocial mechanism, and the perception of community environment affects depression through the mediation of subjective social class. The perception of community public facilities, community living environment, community public security, community neighborhood relationship, community neighborhood mutual assistance, and community emotional attachment significantly affect depression through the mediating role of subjective social class, but the effects are different. The community environment not only has the spatial physical attribute, but also has been endowed with the social psychological effect. The direct effect of community living environment and community public facilities is the biggest, which means that strengthening the optimization and improvement of community physical environment and improving the public’s positive life experience is the primary task. The significant influences of community emotional attachment, community security situation, community neighborhood mutual assistance, and community neighborhood relationship also tell us that strengthening community culture construction, promoting public community emotional social support, and community interpersonal communication are favorable measures to improve the public subjective social class perception and suppress depression.

A contextualized perspective is important to better understand the underlying situational psychosocial mechanisms through which community environment perception has a significant effect on depression. Our results resonate with this point and suggest that the social and cultural psychological background should be considered when studying the impact of community environment on depression. We propose that community environment affects depression through which the mechanism is not a general research problem, but a contextualized problem. Our findings not only improve our understanding of the impacts of community environment on depression, but also have policy implications on how the “healthy China 2030” program related to community environment and people’s well-being. We strongly recommend that the national environmental protection department and civil affairs department should improve the construction of community living environment and public facilities, so as to inspire people’s comfortable experience of community physical living space. Moreover, it is quite necessary to strengthen the construction of community culture to enhance people’s community emotional social support system.

This study makes a preliminary exploration on the social psychological effects of community environment. As the data are cross-sectional data, there is still a lack of dynamic mechanism investigation. It is suggested to carry out a detailed analysis of longitudinal panel data in the future, so as to further obtain reliable conclusions of tracking data.

## Figures and Tables

**Table 1:** The results of the descriptive analysis (*n* = 4997).

**Variables**	**Percentage**	**Variables**	**Percentage**
**Age**		**Nature of Work**	
Age (16–44) = 0	2024 (40.50%)	Agricultural work = 0	2004 (40.90%)
Age (45–59) = 1	1561 (31.20%)	Non-agricultural work = 1	1863 (37.30%)
Age (over 60) = 2	1412 (28.30%)	Jobless = 2	1090 (21.80%)
**Gender**		**Household Register**	
Female = 0	2621(52.50%)	Rural = 0	3685 (73.70%)
Male = 1	2376 (47.50%)	Urban = 1	1312 (26.30%)
**Marital Status**		**Health Condition**	
Unmarried = 0	582 (11.60%)	Unhealthy = 0	1753 (35.10%)
Married with spouse = 1	4021 (80.50%)	Healthy = 1	3244 (64.90%)
Divorce or widowhood = 2	394 (7.90%)		
**Education**			
Primary-level (Illiteracy/Semi-illiterate/Primary) = 0			1413 (28.30%)
Intermediate-level (Junior/Senior/Technical/Professional High School) = 1			2379 (47.60%)
High-level (College/Undergraduate/Master/Doctor) = 2			1205 (24.10%)

**Table 2:** Descriptive statistics and correlation coefficients for the study variables.

	CEP	CPFP	CLEP	CPSP	CNRP	CNMP	CEA	SSC	D
Community Environment Perception (CEP)	1								
Community Public Facilities Perception (CPFP)	0.641 **	1							
Community Living Environment Perception (CLEP)	0.684 **	0.465 **	1						
Community Public Security Perception (CPSP)	0.702 **	0.409 **	0.485 **	1					
Community Neighborhood Relationship Perception (CNRP)	0.656 **	0.242 **	0.271 **	0.344 **	1				
Community Neighborhood Mutual-assistance Perception (CNMP)	0.524 **	0.101 **	0.108 **	0.156 **	0.346 **	1			
Community Emotional Attachment (CEA)	0.602 **	0.168 **	0.202 **	0.238 **	0.356 **	0.370 **	1		
Subjective Social Class (SSC)	0.181 **	0.095 **	0.122 **	0.115 **	0.101 **	0.111 **	0.148 **	1	
Depression (D)	0.216 **	0.110 **	0.106 **	0.144 **	0.164 **	0.161 **	0.148 **	0.107 **	1
M	13.720	2.720	2.770	2.550	2.170	1.510	2.000	3.150	32.350
SD	3.255	0.888	0.914	0.880	0.798	0.793	0.837	1.066	8.045

*N* = 4997, ** *p* < 0.01.

**Table 3:** Linear regression of CEP on depression in different groups.

Variables	Model 1	Model 2	Model 3	Model 4	Model 5	Model 6	Model 7
	Female (*n* = 2621)	Male (*n* = 2376)	Rural (*n* = 3685)	Urban (*n* = 1312)	Age (16–44) (*n* = 2024)	Age (45–59) (*n* = 1561)	Age (Over 60) (*n* = 1412)
CEP	0.479 *** (0.048)	0.597 *** (0.048)	0.561 *** (0.039)	0.516 *** (0.066)	0.611 *** (0.051)	0.655 *** (0.060)	0.515 *** (0.069)
R2	0.036	0.062	0.052	0.045	0.067	0.071	0.038
F	98.325	157.679	202.176	61.776	144.308	118.749	55.150
CPFP	0.984 *** (0.187)	1.034 *** (0.171)	0.944 *** (0.149)	1.024 *** (0.239)	1.214 *** (0.194)	1.418 *** (0.229)	0.779 *** (0.246)
R2	0.010	0.015	0.011	0.014	0.019	0.024	0.007
F	27.698	36.590	40.278	18.284	39.213	38.376	10.040
CLEP	0.797 *** (0.176)	1.121 *** (0.171)	0.962 *** (0.144)	1.020 *** (0.233)	0.916 *** (0.189)	1.378 *** (0.218)	0.814 *** (0.242)
R2	0.008	0.018	0.012	0.014	0.011	0.025	0.008
F	20.407	42.913	44.418	19.195	23.436	40.051	11.344
CPSP	1.103 *** (0.182)	1.543 *** (0.177)	1.389 *** (0.148)	1.047 *** (0.245)	1.647 *** (0.182)	1.738 *** (0.229)	0.913 *** (0.267)
R2	0.014	0.031	0.023	0.014	0.039	0.036	0.008
F	36.864	75.629	87.687	18.307	81.757	57.858	11.700
CNRP	1.567 *** (0.199)	1.791 *** (0.196)	1.736 *** (0.164)	1.484 *** (0.263)	1.594 *** (0.202)	1.957 *** (0.253)	1.757 *** (0.287)
R2	0.023	0.034	0.029	0.024	0.030	0.037	0.026
F	62.050	83.760	111.817	31.934	62.419	59.970	37.613
CNMP	1.590 *** (0.195)	1.598 *** (0.205)	1.845 *** (0.166)	1.360 *** (0.262)	1.474 *** (0.204)	1.776 *** (0.264)	2.053 *** (0.279)
R2	0.025	0.025	0.032	0.020	0.025	0.028	0.037
F	66.810	60.648	123.307	26.903	52.093	45.325	54.216
CEA	1.215 *** (0.193)	1.606 *** (0.185)	1.632 *** (0.157)	1.406 *** (0.258)	1.641 *** (0.191)	1.700 *** (0.246)	1.352 *** (0.276)
R2	0.015	0.031	0.029	0.022	0.035	0.030	0.017
F	39.685	75.444	108.666	29.641	73.714	47.740	23.987

All covariates have been controlled. *** *p* < 0.001.

**Table 4:** Results of hierarchical regression analyses.

Regression Equation		Model Fit Index		Significance of Regression Coefficient			BC 95% CI		DE (IE)
DV	IV	R^2^	F	β	SE	t	LLCI	ULCI	
Depression	CEP	0.168	115.699	0.532	0.036	14.880 ***	0.462	0.602	94.55% (5.45%)
SSC	CEP	0.058	34.452	0.048	0.005	9.668 ***	0.038	0.058	
Depression	CEP	0.174	107.507	0.503	0.036	13.871 ***	0.432	0.574	
	SSC			0.609	0.109	5.573 ***	0.395	0.823	
Depression	CPFP	0.134	88.690	0.931	0.133	7.017 ***	0.671	1.191	92.91% (7.09%)
SSC	CPFP	0.043	25.863	0.085	0.018	4.726 ***	0.050	0.120	
Depression	CPFP	0.144	55.492	0.865	0.132	6.560 ***	0.606	1.123	
	SSC			0.778	0.110	7.050 ***	0.562	0.995	
Depression	CLEP	0.135	56.101	0.929	0.129	7.187 ***	0.676	1.183	90.85% (9.15%)
SSC	CLEP	0.047	27.647	0.112	0.017	6.466 ***	0.078	0.146	
Depression	CLEP	0.144	55.488	0.844	0.129	6.540 ***	0.591	1.097	
	SSC			0.759	0.111	6.825 ***	0.541	0.977	
Depression	CPSP	0.141	95071	1.215	0.133	9.133 ***	0.954	1.476	93.33% (6.67%)
SSC	CPSP	0.046	27.585	0.109	0.018	5.967 ***	0.073	0.145	
Depression	CPSP	0.150	90.296	1.134	0.133	8.555 ***	0.874	1.394	
	SSC			0.745	0.110	6.799 ***	0.530	0.959	
Depression	CNRP	0.146	101.816	1.531	0.143	10.715 ***	1.250	1.811	94.71% (5.29%)
SSC	CNRP	0.044	26.786	0.108	0.020	5.492 ***	0.070	0.147	
Depression	CNRP	0.156	97.876	1.450	0.144	10.084 ***	1.168	1.732	
	SSC			0.740	0.110	6.710 ***	0.524	0.956	
Depression	CNMP	0.147	97.405	1.563	0.149	10.477 ***	1.270	1.855	94.50% (5.50%)
SSC	CNMP	0.045	27.487	0.117	0.022	5.293 ***	0.074	0.160	
Depression	CNMP	0.156	93.087	1.477	0.149	9.891 ***	1.184	1.770	
	SSC			0.733	0.109	6.713 ***	0.519	0.947	
Depression	CEA	0.148	55.208	1.540	0.139	11.047 ***	1.266	1.813	93.18% (6.82%)
SSC	CEA	0.051	31.880	0.150	0.019	8.054 ***	0.114	0.187	
Depression	CEA	0.157	94.024	1.435	0.140	10.260 ***	1.161	1.709	
	SSC			0.697	0.110	6.353 ***	0.482	0.912	

*N* = 4997, dependent variables (DV), independent variables (IV), direct effect (DE), indirect effect (IE). *** *p* < 0.001. All covariates were controlled in the model test.

## Data Availability

Data are available through the Institute of Social Science Survey (ISSS), Peking University, available online website: http://www.isss.pku.edu.cn/ (accessed on 2 June 2021).
